# Alternative *in vitro and in silico* models to reduce animal use: a crucial technological advance in dermatological sciences

**DOI:** 10.1017/erm.2026.10032

**Published:** 2026-01-23

**Authors:** Carla Zanatelli, Luiza Pretto, Thaís Casagrande Paim, Márcia Rosângela Wink

**Affiliations:** 1 Universidade Federal de Ciências da Saúde de Porto Alegre (UFCSPA), Porto Alegre, Brazil; 2 Programa de Pós graduação em Ciências da Saúde; 3 Departamento de Métodos Diagnósticos (DMD); 4 Departamento de Ciências Básicas da Saúde (DCBS)

**Keywords:** alternative models, animal testing, dermatological tests, in vitro skin models, in silico skin models, animal replacement

## Abstract

**Introduction:**

Alternative models are tools to replace and reduce the number of animals used in biomedical sciences, for either research or tests of industrial products. Several new alternative models have been developed in the most diverse fields. Their implementation has led to significant advances in the dermatological cosmetic industry, enabling chemical and molecular screening without animal use. However, limitations remain, particularly regarding tissue microenvironment complexity and systemic metabolic responses.

**Objectives:**

The objective of this viewpoint is to present the existing alternative models available for dermatological sciences, evaluate their applications and discuss their advantages and disadvantages, as well as the future perspectives for safe clinical translation.

**Results:**

In vitro and in silico approaches provide reliable platforms for toxicity, irritation, sensitization, and topical efficacy in cosmetic and dermatological research. Advanced systems, including human skin equivalents, bioprinted skin, and skin-on-a-chip platforms, enhance physiological relevance and mechanistic insight compared with two-dimensional cultures. However, limitations related to tissue complexity, systemic metabolic integration, standardization, and scalability still restrict their ability to fully replace in vivo models.

**Conclusion:**

Therefore, it is expected that future developments in alternative technologies will further enable the reduction of animal model use, while still providing reliable and translatable knowledge applicable across scientific disciplines.

## Introduction

To understand human physiology and pathology, the scientific community in its beginnings used dogs, chickens, rabbits, rodents, pigs, cows, sheep and non-human primates (Ref. 1). It is indisputable that, for the time, the use of these animals was necessary and allowed gaining the wide basic knowledge fundamental for the dissemination of several therapies that currently exist.

However, despite their historical contribution, animal models have limitations inherent in interspecific differences, especially regarding immunological response, gene expression, metabolism and signalling pathways, which often compromises their predictive ability for human outcomes (Refs. 2, 3). It is estimated that more than 90% of drugs that demonstrate efficacy in animal models fail in human clinical trials due to unexpected toxicity or lack of efficacy (Ref. 4). These discrepancies highlight the need for systems more representative of human biology.

Not only studies evoking treatments but also those that aimed to improve the ways of studying went ahead. In this scenario, after some time, alternative models were thought and many continue to be developed to reduce the use of animals, based on the concept of the ‘three Rs’ (replacement, reduction and refinement) created during the 1950s by Russel and Burch (Refs. 5, 6).

Nevertheless, the use of these approaches has not fully replaced animal use yet. Animal testing is still required when it comes to understanding systemic situations, as even with excellent alternative models, the interaction between physiological systems has not been fully replaced to understand the homoeostasis of an organism (Ref. 7). Examples of these situations are studies involving immunological diseases, sepsis and pharmacological action of drugs or chemical substances. In these cases, a scenario emerges that highlights the need to search for new methods that can reverse this situation, drawing attention to the bioethical issue. In addition, the United Nations (UN) established 17 Sustainable Development Goals (SDGs) in 2015, where the promotion of innovation is cited (SDG 9: build resilient infrastructures, promote inclusive and sustainable industrialization and foster innovation).

In this unfilled gap is noticeably visible the emergence of the need to promote more discussions to save animals. It is perceived that there is a worldwide concern, as pharmaceutical companies have been developing cruelty-free products and a growing number of people have adhered to veganism (Ref. 8). According to recent surveys, approximately 1% of Americans and 2% of British adults identify themselves as vegan (Refs. 9, 10). Furthermore, in a study addressing public opinion regarding animal use in medical training, Merkley et al. (2018) found that the majority of the interviewed agree that if effective alternative models exist to study a determined condition, they should be used instead of animals for medical training. A supermajority of these participants agreed that it is unethical or morally wrong to use animals for this reason when non-animal methods are available (Ref. 11).

## Regulatory and ethical advances

Following this time frame, several movements around the world have gained strength to refine ethical issues. In Brazil, a law was created in 2008 to manage animal use: the Arouca Law (11.794/2008). It has established procedures for the scientific use of animals and created the National Council for the Control of Animal Experiments (CONCEA), the highest Brazilian agency in the area. The CONCEA validates alternative methods for testing medicines, cosmetics and health products, among other substances, aiming to replace the use of animals in cosmetic tests. Moreover, each *in vivo* study needs to be approved by a local committee before starting. Not rare, the committee returns the study to the researcher for adjustments, aiming to minimize the number of animals used and their suffering. Similar to Brazil, other countries have their laws and guidelines with the same goal.

**Figure 1.**
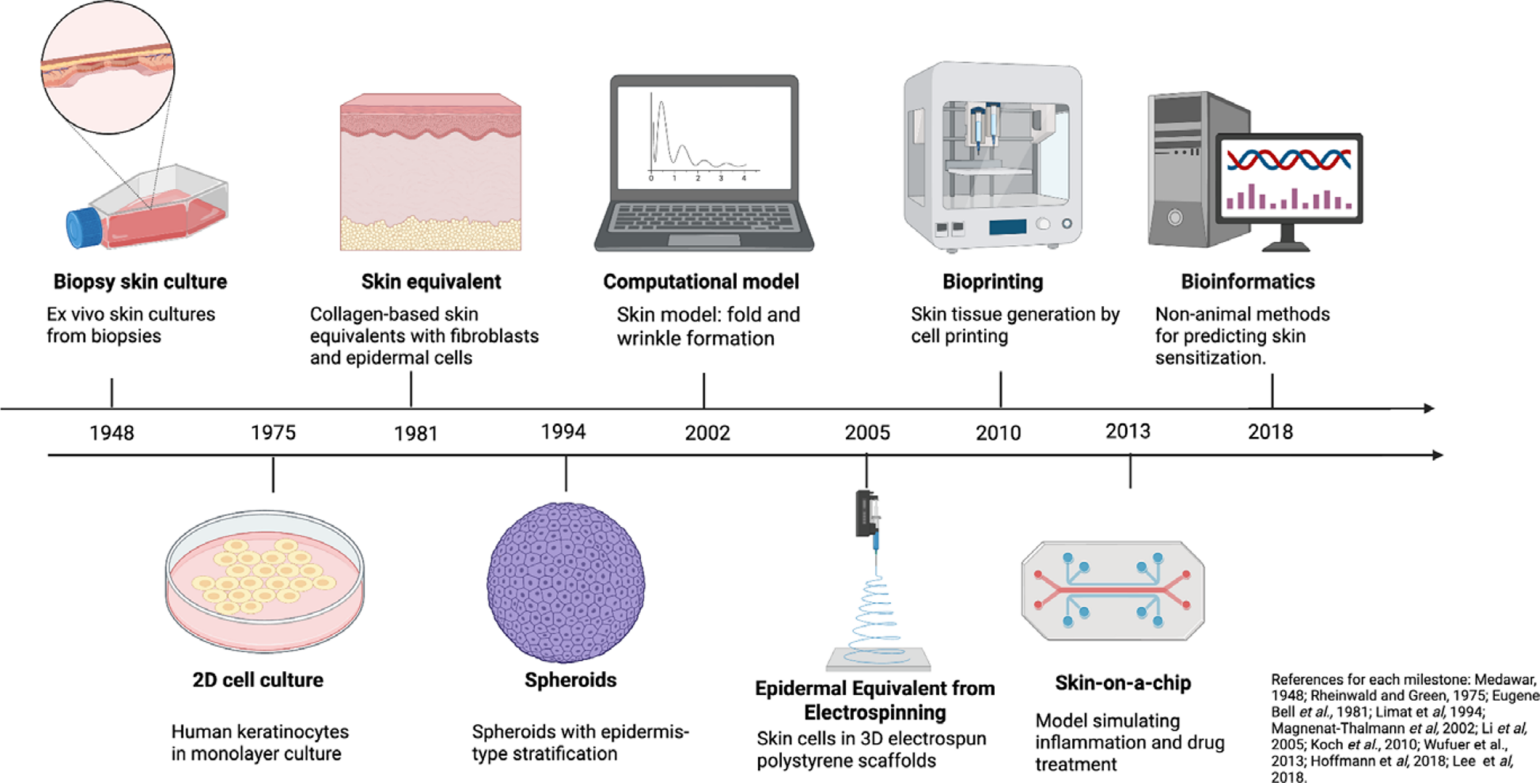
**Timeline showing the emergence of alternative methods.** The figure summarizes the chronological development of key *in vitro* and *in silico* models, from early biopsy and 2D monolayer cultures to advanced 3D bioprinting, skin-on-a-chip and computational systems. Created with BioRender.com.

In an attempt to avoid the use of animals, the development and enforcement of regulations have played a fundamental role in defining ethical standards and research practices regarding the use of animals in science worldwide, as well as in stimulating the development of alternative models and improving the existing ones. In this context, one field that has benefited significantly is dermatology. Considering that dermatology involves the testing of cosmetic substances, most alternative models were developed to meet this need. Alternative *in vitro* and *in silico* models are emerging as a new paradigm, offering tools capable of reproducing aspects of human physiology that animals cannot accurately simulate. While traditional *in vivo* models allow the assessment of systemic responses and interactions between organs, they lack cellular and molecular precision. On the other hand, *in vitro* models, derived from human tissues or cells, provide direct observation of specific human processes – such as gene regulation, protein expression and metabolism – eliminating interspecific variability and increasing the reproducibility of results (Figure 1) (Refs. 12–20).

In the European Union (EU), animal tests for finished cosmetics and cosmetic ingredients were banned in 2004 and 2009, respectively (Ref. 21). In this context, the Scientific Committee on Consumer Safety (SCCS) was created in the EU as an independent committee of experts from different countries. One of its responsibilities is to establish guidelines for cosmetic industries to develop studies for the safety evaluation of cosmetics and relevant toxicological tools for the safety evaluation of cosmetic ingredients as alternatives to animal testing, such as corrosivity and irritation, skin sensitization, dermal/percutaneous absorption, repeated dose toxicity, reproductive toxicity, mutagenicity/genotoxicity, carcinogenicity, toxicokinetics studies, photo-induced toxicity and human data (Ref. 22). The main challenge of overcoming *in vitro* results is to predict the real benefits of cosmetic products in contact with all the systems that involve the human body as a whole (Ref. 23).

**Figure 2.**
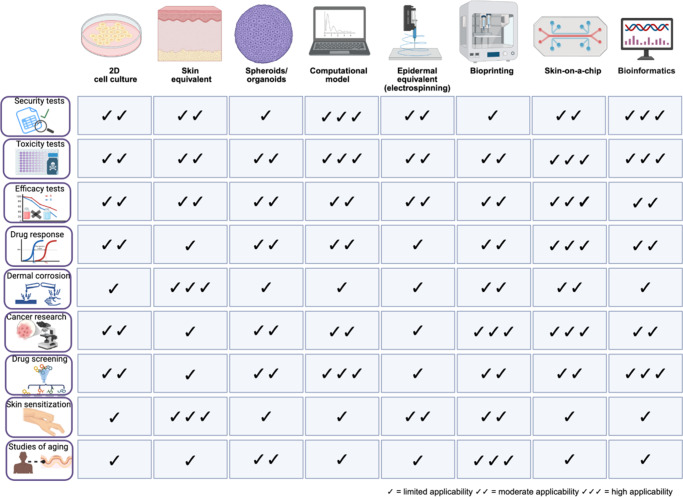
Alternative methods and their correlation with different biological purposes. Created in BioRender. Wink, M. (2026) https://BioRender.com/6v061nr.

The alternative methods to dermatology research have their own advantages and disadvantages (Table 1). A common feature among them is the use as proof of concept, where a large funnel is generated and only substances and/or therapies with pharmacological activity or those with biological potential will proceed to animal testing (Ref. 24). The majority of these alternative methods are cell-based *in vitro* models. Figure 2 summarizes the main alternative dermatological models and their relative applicability to different biological purposes, highlighting differences in suitability, maturity and physiological relevance of each model.

**Table 1.** Main applications, advantages and disadvantages of alternative dermatological methods in substitution for animal tests

Model	Composition	Applications	Advantages	Disadvantages	References
Two-dimensional (2D) monolayer culture	Cells cultured on plastic surface	Cell proliferation and differentiation Gene expression and signalling studies Viral or bacterial infection Transport and uptake models Cancer biology Drug toxicity	Simplicity and cost-effectiveness Straightforward to maintain Control and reproducibility High performance	Cytoskeleton alteration and abnormality in cellular functions, such as metabolism and protein expression No representative cell-to-cell or cell-to-extracellular-matrix interactions Predictivity	(Ref. 25)
Human skin equivalents (HSEs) *or* Human epidermal equivalents (HEEs)	Three-dimensional model using one or more cell types	Assessment of chemical compounds with respect to efficacy and safety Disease modelling	Authenticity Greater physiological relevance Improved predictability and tissue representation Evaluation of compounds after topic exposure	Cost and complexity Standardization and reproducibility Scale limitations Access to nutrients and oxygen Altered *in vitro* morphogenesis Limitation of primary tissue source when using primary cultures of cells	(Ref. 26)
Spheroids	Three-dimensional epithelial cultures consisting of either single-cell lineages or multicellular aggregates arranged into spheroid structures	Drug response measurements Cancer research Regenerative medicine	Self-organization capacity Deposit extracellular matrix Formation of specific microenvironment	Heterogeneity Size limitations Susceptible to central necrosis	(Ref. 27)
Organoids	Cultures composed of various cell types, with mandatory inclusion of epithelial and mesenchymal originated cells	Drug response measurements Organ development research Disease modelling Drug efficacy Skin sensitization	Organotypic phenotype Self-renewal capacity Survival at long term	Low control over cell architecture Slow production Small to mimic microenvironmental tissue conditions Cost and complexity	(Refs. 27–29)
Computational models	Computational numerical models or microarray-based platforms	Tissue design Studies of ageing Skin sensitization Drug efficacy Disease modelling Toxicity analysis Cancer research	Enable rapid prediction generation Cost-effectiveness Control and reproducibility Eliminate the need for physical testing materials Allow simulation of diverse environments and scenarios Complex data analysis	Simplifying biological reality Input data dependence Significant error rates and outcomes can vary considerably Experimental validation required Learning curve Investigators’ biases	(Ref. 15, 30)
Epidermal equivalent from electrospinning	Nylon and primary cells cultured	Dermal irritation/corrosion Formulation efficacy assessment Skin absorption studies	Similarity to the skin barrier Convenient process ability and handling Longer shelf-life Simplified production Low-cost model due to the use of collagen-exempt materials	Lack of biological components Absence of skin appendages Lack of other skin layers Altered biological responses	(Ref. 31)
3D bioprinting	Bioprinted construct made of a bioink containing viable cells	Tissue engineering Regenerative medicine Disease modelling Drug efficacy Drug toxicity	Bioprinted tissue can be constructed entirely from human cells, preventing interspecies-related biases Scalability	Establishment of a vascular network is limited Standardization Cost and complexity	(Ref. 29)
Skin-on-a-chip (SoC)	Bioengineered microdevices with multiple relevant cell types, perfusion and/or biomechanical factors	Toxicity, corrosion and irritation tests Sensitization assessment Efficacy of new compounds and therapeutics	High control over cell architecture Low necessary volume of samples and supplies	Standardization and reproducibility Slow production Cost and complexity	(Ref. 32)
Bioinformatics	*In silico* models using omics data, machine learning, systems biology, molecular dynamics, etc.	Support the analysis, prediction and validation of experimental models Identification of potential target genes	No need for physical samples High-throughput analysis Data integration (multi-omics) Complements *in vitro* models	Requires high-quality datasets Model-dependent outputs Requires experimental validation Limited standardization	(Refs. 33, 34)

## Alternative *in vitro* models in dermatological research

### Two-dimensional (2D) monolayer cultures, spheroids and organoids

Two-dimensional (2D) models are the most commonly used. In adherent 2D cultures, cells grow as an attached monolayer, and the advantages of these cultures are associated with simple and cost-effective maintenance. Unfortunately, adherent cultures have disadvantages as the cultured cells do not mimic the natural tissue structures and, after tissue isolation under 2D conditions, the morphology of the cells is altered as well as the mode of cell division (Ref. 35). Three-dimensional (3D) models that mimic skin tissue have been developed to overcome the limitations of predictivity of 2D *in vitro* models. 3D models showing the interactions between different cell types of tumour and stroma are difficult to generate; thus, to better mimic the *in vivo* situation, different 3D cell culture approaches with several degrees of complexity have been developed. Spheroids have formats that are very useful for basic and applied melanoma research, track cellular behaviour in a cell-type-specific manner and recapitulate different characteristics of early melanoma stages. They can be used to evaluate novel drugs and combination therapies (Ref. 36). Organoids represent an advanced 3D *in vitro* model that mimics the structural and functional organization of native tissues through the self-organization of stem cells or progenitor cells. In dermatological research, skin organoids are capable of reproducing multiple cell types found in the epidermis and dermis, providing a physiologically relevant platform for studying processes such as differentiation, wound healing and disease modelling. These systems offer a more faithful representation of tissue microarchitecture compared to conventional monolayer cultures, bridging the gap between 2D models and full-thickness engineered skin equivalents. Recent studies have demonstrated their potential for applications in drug response testing, toxicity evaluation and personalized medicine approaches in skin biology (Refs. 4, 37, 38).

### Human skin equivalents (HSEs) and reconstructed human epidermis (RHE)

Techniques for developing 3D skin models demand a high level of innovation and complexity through cell cultures. One of the main goals is the *in vitro* development of skin equivalents for dermal toxicity evaluation (Ref. 39). The simplest 3D model is known as reconstructed human epidermis (RHE) and contains an epidermal layer of skin composed only of keratinocytes. EpiSkin™, Epiderm™, SkinEthic™, epiCS™ and LabCyte EPI-MODEL24 are commercially available models of RHE approved by European Centre for the Validation of Alternative Methods (ECVAM) to replace *in vivo* rabbit skin irritation tests and for skin corrosion tests of cosmetic ingredients; more recently, Skin+ and KeraSkin™ have also been validated as irritation tests (Refs. 40, 41). KeratinoSens™ and LuSens are models constructed with immortalized HaCaT stably transfected with a selectable plasmid, which are used in skin sensitization tests also approved by ECVAM (Ref. 42). Full-thickness skin models or HSEs are more complex models, which consist of an epidermis and dermis (keratinocytes and fibroblasts) and have been used to evaluate drug or treatment efficacy (Refs. 43, 44). Recently, 3D vascularized human skin equivalents (vHSEs) that can mimic native skin have been developed; these models reproduce key histological and functional characteristics of real skin, including a multilayered, differentiated epidermis supported by a fibroblast-derived dermal matrix containing endothelial cells organized into a capillary-like microvascular network (Refs. 40, 41). This way, the electrospinning technique has been used to form polymeric mats that, together with porous structures, allow the seeding of cells such as keratinocytes and fibroblasts, to produce an equivalent skin (Ref. 31).

### 3D bioprinting

3D bioprinting is another important tool for dermal toxicity evaluation; it is used to extrude materials and develop scaffolds to target tissues in the required structure (Ref. 45). It enables the accurate construction based on biomolecules, synthetic/natural hydrogels and cells (Refs. 46, 47). Different bioprinting methods have been used to fabricate skin tissue models, such as extrusion-based, laser-assisted and microvalve-based bioprinting (Ref. 45). Authenticity, scalability and reproducibility of the tissues are the main advantages of bioprinted skin, when compared to conventional constructs (Ref. 26). 3D skin bioprinting has enabled the construction of a complete skin with a hypodermal layer (Ref. 48) and can be applied in penetration and absorption tests of cosmetic ingredients. However, there are challenges to overcome, such as producing sensitive, dry, oily skin with different textures, pigmented with different shades, and appendages, hair follicles, microvessels and immune cells (Refs. 26–49). 3D bioprinting is a research target of global cosmetic leaders and still requires standardization and regulatory approvals.

### Skin-on-a-chip (SoC)

Limitations on traditional 2D cultures and 3D organ models have stimulated the use of technologies such as microfabrication in the last decade. In this context, organs-on-chips have been developed. They consist of complex tissue-like structures within microfluidic chips that allow the dynamic culture of cells inside, in order to model or mimic the physiology of a tissue or organ. Skin-on-a-chip (SoC) has made considerable advances in the transport of substances or delivery. Microfluidics maintain the high-throughput capacity of the systems while reducing costs and reagent volumes needed for the experiments (Refs. 50, 51)). In 2D SoC models, skin cells are cultured directly in microfluidic channels to simulate different skin components. Additionally, SoC is a possible platform to study cell–cell interactions, expose cells to mechanical strains or even study immune response (Ref. 52). In 2D SoC systems, monolayer cultures differ from conventional 2D cultures because cells are exposed to dynamic nutrient flow, mechanical stimuli and controlled microenvironments, which help preserve morphology and enable the physiological study of cell–cell interactions. However, 3D SoC models are the most accurate to replicate the spatial organization, cell interactions and functional complexity of native skin, surpassing the limitations of 2D cultures and 2D SoC systems (Refs. 53–55).

Therefore, these models bring human physiology closer than ever, unlike animals, whose physiological parameters (e.g. perfusion rate and oxygen gradients) vary widely. SoC models allow precise control of the cellular microenvironment, reproducing perfusion, mechanical tension and nutrient flow under simulated human conditions (Ref. 56).

## Computational and *in silico* models

Bioinformatics has emerged as a promising alternative to the use of animals and *in vitro* experiments as it allows the investigation of biological phenomena using computational tools and biological databases. Through *in silico* simulations and modelling, it is possible to predict molecular interactions, effects of genetic mutations and drug responses, thus reducing the need for laboratory testing.

The computational models generated from bioinformatics tools are emerging as a new opportunity to reduce not only the use of animal models but also the volume of *in vitro* experiments, contributing to the formulation of more accurate hypotheses before performing biological experiments due to the large volumes of omics data (transcriptomics, proteomics and metabolomics) generated – to the detriment of limited data of traditional tests.

Furthermore, bioinformatics makes it possible to leverage data already available in public databases, enabling comparative and predictive analyses without the need for new experimental tests. This translates into savings in time and resources, as well as greater reproducibility and control over the variables analysed. Thus, scientific research becomes more sustainable and aligned with ethical principles that seek to replace, reduce and refine the use of animals in experimentation.

Utilizing bioinformatics tools and *in silico* experiments, data from human cell cultures exposed to different toxicants can be analysed (e.g. (Ref. 57)). Interestingly, dermatology was one of the first medical disciplines to welcome and support bioinformatics results, and the term ‘skinomics’ has been proposed to designate specifically the bioinformatics studies in the area. The objective is to provide knowledge of skin biology, improve the function of healthy skin and assist in treating pathological skin conditions through *in silico* experiments, where computer simulations using databases allow the modelling of an organic phenomenon (Refs. 58–60). *In silico* models make predictions concerning absorption, distribution, metabolism and excretion of a chemical from molecular data, and new possibilities and results that can be generated by this approach are promising and exciting (Refs. 58–60).

More recently, researchers are exploring the uses of artificial intelligence (AI) to improve or supplement current screening processes in melanoma and nonmelanoma skin cancer (Ref. 60). These methods can be explored in the future for use as strategic alternatives to animal research as well. In the context of dermatological and cosmetic sciences, AI can be trained on databases containing chemical descriptors, gene-expression signatures and cellular responses from reconstructed human skin models to predict irritation, sensitization, penetration and antimicrobial efficacy of novel compounds before physical testing. These approaches not only accelerate early-stage screening but also substantially reduce the number of compounds entering animal experimentation, thereby minimizing animal use and costs (Ref. 61).

**Figure 3.**
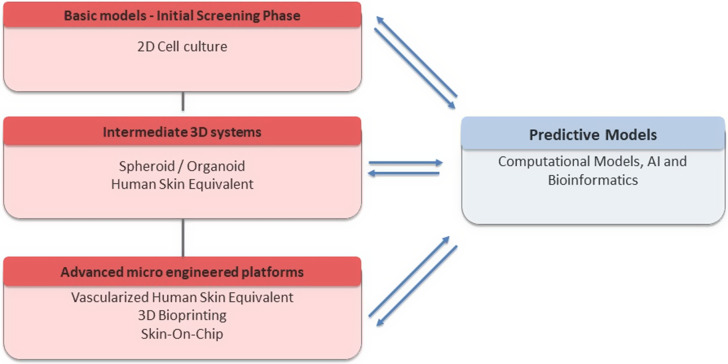
Flowchart illustrating the progressive transition from basic to advanced animal-free dermatological models and their integration with predictive models.

Despite their clear advantages, AI and other computational approaches still rely on robust, high-quality datasets – often derived from *in vivo*, *in vitro* and clinical studies – to ensure the reliability and generalizability of their predictions (Ref. 20). In this context, bioinformatics plays a central role in data analysis, model prediction and the refinement of experimental designs, establishing itself as an indispensable tool in modern science. It contributes to the integration of technological innovation with ethical and sustainable research practices (Figure 3). Nonetheless, computational predictions still require experimental validation to confirm their biological relevance and applicability. Therefore, additional time, resources and continued investment will be necessary before such tools can meaningfully reduce, though not fully replace, the use of traditional *in vitro* and *in vivo* models.

## Conclusion and perspectives

The alternative models cited represent a valuable strategy to mimic biological systems and minimize animal use. A number of models and techniques have been developed for different purposes. 2D monolayer cultures remain indispensable for hypothesis generation and screening studies due to their simplicity and cost-effectiveness. However, by design, they lack the 3D architecture and microenvironment of native skin. As a result, 2D systems cannot simulate complex tissue responses and should be complemented by advanced 3D models, microphysiological platforms and *in silico* approaches when higher physiological relevance is required.

Despite their potential, advanced methods are not yet widely adopted across research settings. It is notable that more developed countries are already ahead in the use of these methodologies. However, these innovative solutions must reach all countries, enabling them to refine and rethink the use of animals in dermal pharmacology and toxicology research and progressively in other fields.

Encouragingly, the latest ECVAM status report indicates that in 2022, approximately 8.5 million animals were used for scientific purposes across the EU, representing a 5% reduction compared to 2018, coinciding with a rapid expansion of *in vitro* and *in silico* biotechnologies (Ref. 62). These trends demonstrate that while complete replacement remains challenging, significant advances are being achieved.

Altogether, we can conclude that there is no unique methodology capable of replacing *in vivo* experiments. It is necessary that each researcher evaluate the scientific question that should be addressed and then select and develop the best *in silico* and *in vitro* protocols or their combination. After that, *in vivo* experimentation needs to be done for the questions that remain to be answered at the systemic level – for example, modulation of the immune system, pharmacokinetics, pharmacodynamics, chronic toxicity – until new possibilities and technologies arise.

## Data Availability

No datasets were generated or analysed during the study.

## References

[1] Franco NH (2013) Animal experiments in biomedical research: A historical perspective. Animals: An Open Access Journal from MDPI 3, 238–273. 26487317 10.3390/ani3010238 PMC4495509

[2] van der Worp HB, Howells DW, Sena ES, Porritt MJ, Rewell S, O’Collins V and Macleod MR (2010) Can animal models of disease reliably inform human studies? PLoS Medicine 7, e1000245. 20361020 10.1371/journal.pmed.1000245 PMC2846855

[3] Hartung T (2008) Thoughts on limitations of animal models. Parkinsonism & Related Disorders 14(Suppl 2), S81–S83. 18585949 10.1016/j.parkreldis.2008.04.003

[4] Mak IW, Evaniew N and Ghert M (2014) Lost in translation: Animal models and clinical trials in cancer treatment. American Journal of Translational Research 6, 114–118. 24489990 PMC3902221

[5] Russell WMS and Burch RL (1959) The Principles of Humane Experimental Technique. London: Methuen.

[6] Doke SK and Dhawale SC (2015) Alternatives to animal testing: A review. Saudi Pharmaceutical Journal: SPJ: The Official Publication of the Saudi Pharmaceutical Society 23, 223–229. 26106269 10.1016/j.jsps.2013.11.002 PMC4475840

[7] Barré-Sinoussi XMF and Montagutelli X (2015) Animal models are essential to biological research: Issues and perspectives. Future Science OA 1(4): FSO63. 10.4155/fso.15.63. 28031915 PMC5137861

[8] Wang Y, Zhao Y and Song F (2020) The ethical issues of animal testing in cosmetics industry. Humanities and Social Sciences 8, 112.

[9] Jones JM (2023) In U.S., 4% Identify as Vegetarian, 1% as Vegan. Gallup. Available at https://news.gallup.com/poll/510038/identify-vegetarian-vegan.aspx (accessed 15 October 2025).

[10] Mathieu E and Ritchie H (2022) What share of people say they are vegetarian, vegan, or flexitarian? *Our World in Data.* Available at https://ourworldindata.org/vegetarian-vegan (accessed 15 October 2025).

[11] Merkley R, Pippin JJ and Joffe AR (2018) A survey to understand public opinion regarding animal use in medical training. Alternatives to Laboratory Animals: ATLA 46, 133–143. 30022674 10.1177/026119291804600308

[12] Rheinwald JG and Green H (1975) Serial cultivation of strains of human epidermal keratinocytes: The formation of keratinizing colonies from single cells. Cell 6, 331–343. 1052771 10.1016/s0092-8674(75)80001-8

[13] Bell E, Ehrlich HP, Buttle DJ and Nakatsuji T (1981) Living tissue formed in vitro and accepted as skin-equivalent tissue of full thickness. Science 211, 1052–1054. 7008197 10.1126/science.7008197

[14] Limat A, Breitkreutz D, Hunziker T, Klein CE, Noser F, Fusenig NE and Braathen LR (1994) Outer root sheath (ORS) cells organize into epidermoid cyst-like spheroids when cultured inside Matrigel: A light-microscopic and immunohistological comparison between human ORS cells and interfollicular keratinocytes. Cell and Tissue Research 275, 169–176. 7509722 10.1007/BF00305384

[15] Magnenat-Thalmann N, Kalra P, Lévêque JL, Bazin R, Batisse D and Querleux B (2002) A computational skin model: Fold and wrinkle formation. IEEE Transactions on Information Technology in Biomedicine: A Publication of the IEEE Engineering in Medicine and Biology Society 6, 317–323. 15224846 10.1109/titb.2002.806097

[16] Li M, Mondrinos MJ, Gandhi MR, Ko FK, Weiss AS and Lelkes PI (2005) Electrospun protein fibers as matrices for tissue engineering. Biomaterials 26, 5999–6008. 15894371 10.1016/j.biomaterials.2005.03.030

[17] Wufuer M, Lee G, Hur W, Jeon B, Kim BJ, Choi TH and Lee S (2016) Skin-on-a-chip model simulating inflammation, edema and drug-based treatment. Scientific Reports 6, 37471. 27869150 10.1038/srep37471 PMC5116589

[18] Hoffmann S, Kleinstreuer N, Alépée N, Allen D, Api AM, Ashikaga T, Clouet E, Cluzel M, Desprez B, Gellatly N, Goebel C, Kern PS, Klaric M, Kühnl J, Lalko JF, Martinozzi-Teissier S, Mewes K, Miyazawa M, Parakhia R, van Vliet E, Zang Q and Petersohn D (2018) Non-animal methods to predict skin sensitization (I): The cosmetics Europe database. Critical Reviews in Toxicology 48, 344–358. 29474128 10.1080/10408444.2018.1429385

[19] Koch L, Kuhn S, Sorg H, Gruene M, Schlie S, Gaebel R, Polchow B, Reimers K, Stoelting S, Ma N, Vogt PM, Steinhoff G and Chichkov B (2010) Laser printing of skin cells and human stem cells. Tissue Engineering. Part C, Methods 16, 847–854. 19883209 10.1089/ten.TEC.2009.0397

[20] Gangwal A and Lavecchia A (2025) Correction to “artificial intelligence in natural product drug discovery: Current applications and future perspectives. Journal of Medicinal Chemistry 68, 14127. 39916476 10.1021/acs.jmedchem.4c01257 PMC11874025

[21] Ban on Animal Testing (n.d.) Available at https://ec.europa.eu/growth/sectors/cosmetics/ban-animal-testing_pt (accessed 9 December 2021).

[22] The SCCS Notes of Guidance for the Testing of Cosmetic Ingredients and their Safety Evaluation – 12th Revision (2023) Luxembourg: Publications Office of the European Union.

[23] Eberlin S, da Silva MS, Facchini G, da Silva GH, Pinheiro ALTA, Eberlin S and Pinheiro A d S (2020) The skin model as an alternative tool for the efficacy and safety evaluation of topical products. Alternatives to Laboratory Animals: ATLA 48, 10–22. 32496151 10.1177/0261192920914193

[24] Denayer T, Stöhr T and Van Roy M (2014) Animal models in translational medicine: Validation and prediction. New Horizons in Translational Medicine 2, 5–11.

[25] Kamatar A, Gunay G and Acar H (2020) Natural and synthetic biomaterials for engineering multicellular tumor spheroids. Polymers 12(11): 2506. 10.3390/polym12112506. 33126468 PMC7692845

[26] Millás A, Lago J, Vasquez-Pinto L, Massaguer P and Maria-Engler SS (2019) Approaches to the development of 3d bioprinted skin models: The case of natura cosmetics. International Journal of Advances in Medical Biotechnology – IJAMB 2, 03. 10.25061/2595-3931/ijamb/2019.v2i1.24.

[27] Sakalem ME, De Sibio MT, da Costa FA d S and de Oliveira M (2021) Historical evolution of spheroids and organoids, and possibilities of use in life sciences and medicine. Biotechnology Journal 16, e2000463. 33491924 10.1002/biot.202000463

[28] Low LA, Mummery C, Berridge BR, Austin CP and Tagle DA (2021) Organs-on-chips: Into the next decade. Nature Reviews. Drug Discovery 20, 345–361. 32913334 10.1038/s41573-020-0079-3

[29] Hagenbuchner J, Nothdurfter D and Ausserlechner MJ (2021) 3D bioprinting: Novel approaches for engineering complex human tissue equivalents and drug testing. Essays in Biochemistry 65, 417–427. 34328185 10.1042/EBC20200153 PMC8365325

[30] Wilm A, Kühnl J and Kirchmair J (2018) Computational approaches for skin sensitization prediction. Critical Reviews in Toxicology, 48(9): 738–760. 10.1080/10408444.2018.1528207. 30488745

[31] Camarena DEM, Matsuyama LSAS, Maria-Engler SS and Catalani LH (2020) Development of epidermal equivalent from electrospun synthetic polymers for in vitro irritation/corrosion testing. Nanomaterials 10, 2528. 33339410 10.3390/nano10122528 PMC7766501

[32] Hardwick RN, Betts CJ, Whritenour J, Sura R, Thamsen M, Kaufman EH and Fabre K (2020) Drug-induced skin toxicity: Gaps in preclinical testing cascade as opportunities for complex in vitro models and assays. Lab on a Chip 20, 199–214. 31598618 10.1039/c9lc00519f

[33] Weng T, Zhang X, He J, Yang Y and Li C (2024) Bioinformatics-based analysis of the relationship between plasminogen regulatory genes and photoaging. Journal of Cosmetic Dermatology 23, 2270–2278. 38634239 10.1111/jocd.16266

[34] Bang H, Kim JE, Lee HS, Park SM, Park D-J and Lee EJ (2022) Integrated bioinformatic analysis of gene expression profiling data to identify combinatorial biomarkers in inflammatory skin disease. Scientific Reports 12, 5889. 35393522 10.1038/s41598-022-09840-3 PMC8989986

[35] Kapałczyńska M, Kolenda T, Przybyła W, Zajączkowska M, Teresiak A, Filas V, Ibbs M, Bliźniak R, Łuczewski Ł and Lamperska K (2018) 2D and 3D cell cultures – A comparison of different types of cancer cell cultures. Archives of Medical Science: AMS 14, 910–919. 30002710 10.5114/aoms.2016.63743 PMC6040128

[36] Vörsmann H, Groeber F, Walles H, Busch S, Beissert S, Walczak H and Kulms D (2013) Development of a human three-dimensional organotypic skin-melanoma spheroid model for in vitro drug testing. Cell Death & Disease 4, e719. 23846221 10.1038/cddis.2013.249 PMC3730422

[37] Lee J, Bӧscke R, Tang P-C, Hartman BH, Heller S and Koehler KR (2018) Hair follicle development in mouse pluripotent stem cell-derived skin Organoids. Cell Reports 22, 242–254. 29298425 10.1016/j.celrep.2017.12.007 PMC5806130

[38] Kim J, Koo B-K and Knoblich JA (2020) Human organoids: Model systems for human biology and medicine. Nature Reviews. Molecular Cell Biology 21, 571–584. 32636524 10.1038/s41580-020-0259-3 PMC7339799

[39] do Nascimento Pedrosa T, Catarino CM, Pennacchi PC, de Moraes Barros SB and Maria-Engler SS (2021) Skin equivalent models: Protocols for in vitro reconstruction for dermal toxicity evaluation. Methods in Molecular Biology 2240, 31–41. 33423224 10.1007/978-1-0716-1091-6_3

[40] OECD (2021) Test No. 439: in vitro Skin Irritation: Reconstructed Human Epidermis Test Method. Available at: 10.1787/9789264242845-en (accessed 20 December 2024).

[41] OECD (2019) Test No. 431: in vitro Skin Corrosion: Reconstructed Human Epidermis (RHE) Test Method. Available at: 10.1787/9789264264618-en (accessed 20 December 2024).

[42] OECD (2022) Test No. 442D: in vitro Skin Sensitisation. Available at: 10.1787/9789264229822-en (accessed 20 December 2024).

[43] Bataillon M, Lelièvre D, Chapuis A, Thillou F, Autourde JB, Durand S, Boyera N, Rigaudeau A-S, Besné I and Pellevoisin C (2019) Characterization of a new reconstructed full thickness skin model, T-skin™, and its application for investigations of anti-aging compounds. International Journal of Molecular Sciences 20(9): 2240. 10.3390/ijms20092240. 31067675 PMC6540298

[44] Mathes SH, Ruffner H and Graf-Hausner U (2014) The use of skin models in drug development. Advanced Drug Delivery Reviews 69–70, 81–102. 24378581 10.1016/j.addr.2013.12.006

[45] Ng WL and Yeong WY (2019) The future of skin toxicology testing – three-dimensional bioprinting meets microfluidics. International Journal of Bioprinting 5, 237. 32596546 10.18063/ijb.v5i2.1.237 PMC7310273

[46] Fayyazbakhsh F (2020) A brief review on 3D bioprinted skin substitutes. Procedia Manufacturing 48, 790–796.

[47] Manita PG, Garcia-Orue I, Santos-Vizcaino E, Hernandez RM and Igartua M (2021) 3D bioprinting of functional skin substitutes: From current achievements to future goals. Pharmaceuticals 14(4): 362. 10.3390/ph14040362. 33919848 PMC8070826

[48] Moakes RJA, Senior JJ, Robinson TE, Chipara M, Atansov A, Naylor A, Metcalfe AD, Smith AM and Grover LM (2021) A suspended layer additive manufacturing approach to the bioprinting of tri-layered skin equivalents. APL Bioengineering 5, 046103. 34888433 10.1063/5.0061361 PMC8635740

[49] Olejnik A, Semba JA, Kulpa A, Dańczak-Pazdrowska A, Rybka JD and Gornowicz-Porowska J (2022) 3D bioprinting in skin related research: Recent achievements and application perspectives. ACS Synthetic Biology 11, 26–38. 34967598 10.1021/acssynbio.1c00547 PMC8787816

[50] Zhang Q, Sito L, Mao M, He J, Zhang YS and Zhao X (2018) Current advances in skin-on-a-chip models for drug testing. Microphysiological Systems 2: 4. 10.21037/mps.2018.08.01. 33521629 PMC7842276

[51] Risueño I, Valencia L, Jorcano JL and Velasco D (2021) Skin-on-a-chip models: General overview and future perspectives. APL Bioengineering 5(3): 031503. 10.1063/5.0046376. 34258497 PMC8270645

[52] Zoio P and Oliva A (2022) Skin-on-a-Chip Technology: Microengineering physiologically relevant in vitro skin models. Pharmaceutics 14(3): 682. 10.3390/pharmaceutics14030682. 35336056 PMC8955316

[53] Zhang B, Korolj A, Lai BFL and Radisic M (2018) Advances in organ-on-a-chip engineering. Nature Reviews. Materials 3, 257–278.

[54] Fernandez-Carro E, Angenent M, Gracia-Cazaña T, Gilaberte Y, Alcaine C and Ciriza J (2022) Modeling an optimal 3D skin-on-Chip within microfluidic devices for pharmacological studies. Pharmaceutics 14(7): 1417. 10.3390/pharmaceutics14071417. 35890312 PMC9316928

[55] Abdo D, Zhao Y, Okhovatian S, Vargas LFJ, Wagner KT, Shakeri A, Vosoughi D and Radisic M (2025) A dermis-on-a-chip model for compound screening. Materials Today. Bio 34, 102111. 10.1016/j.mtbio.2025.102111 PMC12309271 40740248

[56] Rimal R, Marquardt Y, Nevolianis T, Djeljadini S, Marquez AB, Huth S, Chigrin DN, Wessling M, Baron JM, Möller M and Singh S (2021) Dynamic flow enables long-term maintenance of 3-D vascularized human skin models. Applied Materials Today 25, 101213.

[57] Grafström RC, Nymark P, Hongisto V, Spjuth O, Ceder R, Willighagen E, Hardy B, Kaski S and Kohonen P (2015) Toward the replacement of animal experiments through the bioinformatics-driven analysis of “Omics” Data from human cell cultures. Alternatives to Laboratory Animals: ATLA 43, 325–332. 26551289 10.1177/026119291504300506

[58] Younis S, Shnayder V and Blumenberg M (2016) Application of bioinformatics methodologies in the fields of skin biology and dermatology. In: Maglaveras NG and Chouvarda I (eds), Bioinformatics – Updated Features and Applications. London, UK: InTech.

[59] Gunia-Krzyżak A, Popiol J and Marona H (2016) Melanogenesis inhibitors: Strategies for searching for and evaluation of active compounds. Current Medicinal Chemistry 23, 3548–3574. 27356545 10.2174/0929867323666160627094938

[60] Madden JC, Enoch SJ, Paini A and Cronin MTD (2020) A review of in Silico tools as alternatives to animal testing: Principles, resources and applications. Alternatives to Laboratory Animals 48, 146–172. 10.1177/0261192920965977. 33119417

[61] Tieghi RS, Moreira-Filho JT, Martin H-J, Wellnitz J, Otoch MC, Rath M, Tropsha A, Muratov EN and Kleinstreuer N (2024) A novel machine learning model and a web portal for predicting the human skin sensitization effects of chemical agents. Toxics 12(11): 803. 10.3390/toxics12110803. 39590983 PMC11598222

[62] Zuang V, Baccaro M, Barroso J, Berggren E, Bopp S, Bridio S, Capeloa T, Carpi D, Casati S, Chinchio E, Corvi R, Deceuninck P, Franco A, Gastaldello A, Katsanou E, Langezaal I, Malinowska J, Mennecozzi M, Milcamps A, Piergiovanni M, Prieto-Peraita P, Selfa AL, Valsesia D, Whelan M, Wittwehr C and Worth A (2025) *Non-Animal Methods in Science and Regulation.* Luxembourg: Publications Office of the European Union.

